# Highest Occurring Vascular Plants from Ladakh Provide Wood Anatomical Evidence for a Thermal Limitation of Cell Wall Lignification

**DOI:** 10.1111/pce.15221

**Published:** 2024-10-24

**Authors:** Ulf Büntgen, Veronika Jandova, Jiri Dolezal

**Affiliations:** ^1^ Department of Geography University of Cambridge Cambridge UK; ^2^ Global Change Research Institute (CzechGlobe), Czech Academy of Sciences Brno Czech Republic; ^3^ Department of Geography, Faculty of Science Masaryk University Brno Czech Republic; ^4^ Institute of Botany, Czech Academy of Sciences Pruhonice Czech Republic; ^5^ Department of Botany, Faculty of Science University of South Bohemia Ceske Budejovice Czech Republic

**Keywords:** alpine ecology, dendrochronology, plant growth, Tibetan Plateau, treeline research, wood anatomy

## Abstract

As an evolutionary achievement of almost all terrestrial plants, lignin biosynthesis is essential for various mechanical and physiological processes. Possible effects of plant cell wall lignification on large‐scale vegetation distribution are, however, not yet fully understood. Here, we present double‐stained, wood anatomical stem measurements of 207 perennial herbs (*Potentilla pamirica* Wolf), which were collected between 5550 and 5850 m asl on the north‐western Tibetan Plateau in Ladakh, India. We also measured changes in situ root zone and surface air temperatures along the sampling gradient and applied piecewise structural equation models to assess direct and indirect relationships between the age and size of plants, the degree of cell wall lignification in their stems, and the elevation at which they were growing. Based on the world's highest‐occurring vascular plants, the Pamir Cinquefoils, we demonstrate that the amount of lignin in the secondary cell walls decreases significantly with increasing elevation (*r* = −0.73; *p* < 0.01). Since elevation is a proxy for temperature, our findings suggest a thermal constrain on lignin biosynthesis at the cold range limit of woody plant growth.

## Summary statement

Spanning 5550–5850 m asl on the Tibetan Plateau, the degree of cell wall lignification in 207 *Potentilla pamirica* Wolf decreases significantly with increasing elevation. Our finding suggests a thermal control on the lignin biosynthesis pathway at the cold range limit of woody plant growth.

## 1 Introduction

As an evolutionary achievement of almost all terrestrial plants around 460 million years ago (Weng and Chapple 2010; Niklas, Cobb, and Matas 2017), lignin biosynthesis is essential for a range of mechanical and physiological processes (Zhong and Ye 2009; Renault, Werck‐Reichhart, and Weng 2019). Without cell wall lignification, larger plants could not grow tall and upright, conduct water over long vertical distances, resist drought stress, and defend themself against biotic and abiotic stressors (Piquemal et al. 1998; Moura et al. 2010; Schenk et al. 2018; Polo et al. 2020). Lignin is polymerised from monolignols synthesised through the lignification biosynthetic pathway (Zhong and Ye 2009; Barros et al. 2015; Hao and Mohnen 2014; Liu, Luo, and Zheng 2018). As a crucial part of xylogenesis in all plant species with vascular cambium, different lignin compounds can vary within and between wood tissues and cell types and are usually formed after cell enlargement but before cell death (Meents, Watanabe, and Samuels 2018; Kumar, Campbell, and Turner 2016; Mellerowicz et al. 2001). Despite its widespread occurrence and structural function, the possible role lignin plays in biomechanics and macroecology is still under debate (Crivellaro et al. 2022; Büntgen et al. 2023; Körner, Fajardo, and Hiltbrunner 2023). It further remains unclear how cold temperatures may possibly, directly or indirectly, impact the formation and deposition of cellulose, hemicellulose, polysaccharides, and lignin (Begum et al. 2013; Plomion, Leprovost, and Stokes 2001; Rossi et al. 2007), with the latter being the second most abundant plant biopolymer on Earth (after cellulose).

Since von Humboldt's pioneering treeline studies at Chimborazo volcano in Ecuador more than 200 years ago (von Humboldt and Bonpland 1805), biologists, ecologists and geographers have considered various aspects of cell formation, plant growth and vegetation composition to explain global treeline positions (Körner 2012). The putative inability of plants to fully lignify their secondary cell walls under too cold growing season temperatures has been introduced recently as an additional biochemical and biomechanical, rather than an alternative physiological concept to help explain the elevational and latitudinal positions of undisturbed treeline ecotones (Crivellaro et al. 2022; Crivellaro and Büntgen 2020). This hypothesis has been supported by unique time‐for‐space surrogates: so‐called Blue Rings (Piermattei et al. 2015). These newly discovered wood anatomical anomalies refer to a lack of cell wall lignification across entire (very rare) or partial (more frequent) tree rings (Piermattei et al. 2020; Greaves et al. 2023). Blue Rings are sporadically formed when the growing season is affected by ephemeral cold spells that often follow large, sulphur‐rich volcanic eruptions (Piermattei et al. 2020; Büntgen et al. 2022). Since experimental evidence for temperature‐induced disruptions of the biochemical process of cell wall lignification is rare (Büntgen, 2023; Körner, Lenz, and Hoch 2023), open‐field and controlled laboratory experiments are needed to explore the idea of thermal controls on biosynthesis and their possible biogeographic implications.

**Figure 1:**
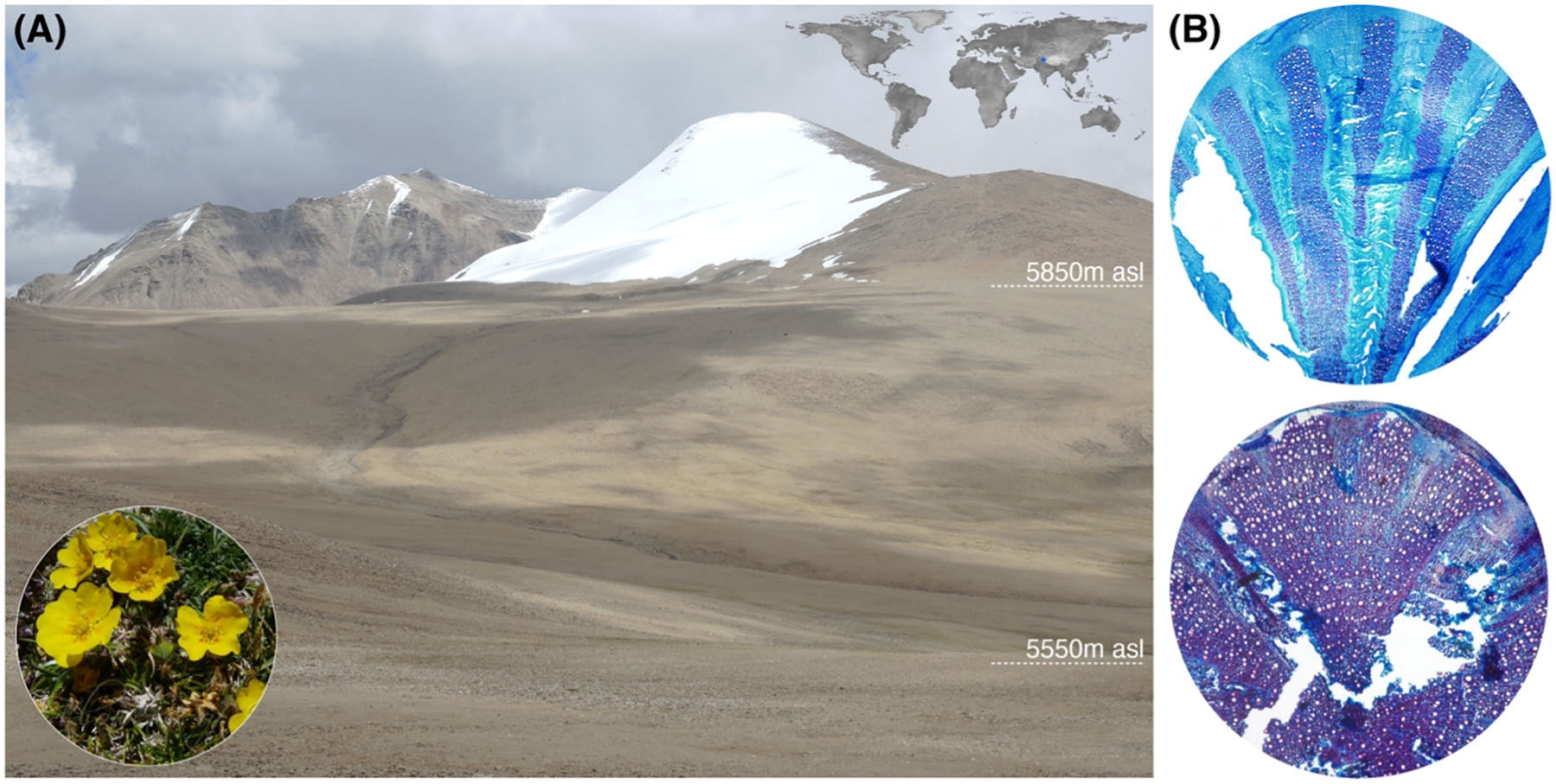
Study region near Chamser Kangri (6622 m asl) in eastern Ladakh (India), north‐western Himalaya. (A) Sample gradient between 5550 and 5850 m asl where 207 plants of the perennial woody herb *Potentilla pamirica* Wolf were excavated in August 2016. The upper right inset places the study site in a global context, while the bottom‐left inset shows a flowering specimen. (B) Wood anatomical thin sections of *Potentilla* from the lower and upper range of the sampling gradient exhibit higher and lower degrees of cell wall lignification (DCWL), respectively (see Supporting Information: Figure S2 for ring boundaries and Supporting Information: Figure S3 for more examples).

Here, we argue that reasons for the absence of trees above the treeline cannot be found in trees below the treeline. Instead, we suggest investigation of smaller plants at higher elevations where surface air and root zone temperatures are too cold for upright tree growth. We therefore collected 207 specimens of the alpine herb (*Potentilla pamirica* Wolf) at five elevational zones between 5550 and 5850 m asl on the south‐eastern slope of the Chamser Kangri (6622 m asl) in eastern Ladakh (Figure 1A and Supporting Information: Figure S1), India (Dolezal et al. 2021a, 2021b). Known as Pamir Cinquefoils and sought‐after since ancient times for its curative properties (Tomczyk and Latté 2009), this woody forb is amongst the world's highest‐occurring vascular plants. The perennial, non‐clonal herbs of the Rosaceae family usually grow 5–15 cm tall, have deep tap roots of 15–20 mm diameter, and form pinnate leaves and yellow flowers (Figure 1A). The species frequently occurs on mesic stony slopes and dry gravel within the subnival zone of Eurasia's high mountain systems, such as the Altai, Himalaya, Karakorum, Pamir and Tien Shan (Supporting Information: Figure S1).

State‐of‐the‐art dendrochronological and wood anatomical techniques were combined to define the age and degree of cell wall lignification (DCWL) in all specimens. We further measured surface air and root zone temperatures at 15‐min resolution from August 2015 to September 2017 along the elevational gradient. Piecewise structural equation models were applied to account for co‐variability and quantify possible relationships between elevation, as well as the age, size and DCWL of all samples.

## 2 Materials and Methods

Our study region east of lake Tso Moriri (34°45′ N and 77°35′ E), and between the peaks of Shukule (6514 m asl) and Chamser Kangri (6622 m asl), is part of the westernmost spur of the Tibetan Plateau in Ladakh, India (Figure 1 and Supporting Information: Figure S1). The snow line around 6100–6200 m asl coincides with the highest recorded occurrence of vascular plants (Angel et al. 2016). Characterised by sparse subnival vegetation (Dvorský et al. 2011) and no impacts by humans and/or grazing animals, we defined five sampling sites along an elevational gradient between 5550 and 5850 m asl. Each site is located on the same slope with similar soil conditions based on coarse‐grained gneiss and high percentages of large gravel. Water and organic matter contents are low, whereas the pH level and macronutrients are high (Řeháková et al. 2017). TOMST TMS3 microclimatic stations were operating from August 2013 to September 2015 at the lower and upper ends of the elevational gradient to record both surface air and root zone temperatures, as well as volumetric soil moisture at 15‐min resolution. Annual precipitation data were obtained from the high‐resolution TerraClimate data set (Abatzoglou et al. 2018).

In August 2016, we collected 207 specimens of the alpine herb *P. pamirica* Wolf at five elevational zones around 5550, 5630, 7700, 5800 and 5850 m asl (Figure 1 and Table 1). The complete above‐ and belowground organs of each plant were excavated. The dry weight of each herb, including roots, stems, leaves, and flowers was used as an estimate for plant size. Shoot and root lengths were measured, and cross sections were cut from the oldest stem tissues between the hypocotyl and primary root (i.e., root collar) using a sledge microtome (Gärtner et al. 2015). With an average thickness of 20–30 µm, all anatomical stem sections were double‐stained with a 1:1 blend of freshly prepared Safranin (1% in water) and Astra Blue (0.5% in water with 0.2% acetic acid), which is best known to reliably visualise lignin red and cellulose blue (Crivellaro et al. 2022; Crivellaro and Büntgen 2020; Piermattei et al. 2015, 2020; Greaves et al. 2023; Büntgen et al. 2022; Büntgen, 2023). All cross‐sections were then dehydrated, washed, and finally fixed in Canada balsam under cover glasses before their optical examination with an Olympus BX53 microscope equipped with an Olympus DP73 camera and cellSense Entry 1.9 software. Plant ages were defined by carefully counting the annual growth rings along two radii on each root collar cross‐section. To estimate the DCWL in the stems of all sample, polygons were placed over each cross‐section to include all tissue types identified by their respective colours (blue = parenchyma, white = conduits, and red = lignin). The area percentages of all three tissue types were quantified using ImageJ software by randomly placing 100 probes within each polygon. The DCWL was then calculated by dividing the lignified cell wall area by the total cell wall area [DCWL = lignified cell wall area (red)/total cell wall area (red and blue)] (Crivellaro et al. 2022). Although simple, the applied double‐staining technique is a well‐established procedure in both quantitative wood anatomy and modern dendrochronology (Crivellaro et al. 2022; Crivellaro and Büntgen 2020; Piermattei et al. 2015, 2020; Greaves et al. 2023; Büntgen et al. 2022; Büntgen, 2023), which reliably visualises lignified cell walls red and cellulose blue (Büntgen et al. 2015; Bond et al. 2008; Baldacci‐Cresp et al. 2020; Srebotnik and Messner 1994; Vazquez‐Cooz and Meyer 2002; De Micco and Aronne 2007).

**Table 1:** Dendrochronological, morphological and anatomical characteristics of *Potentilla pamirica* Wolf. Number of plants collected at five elevational zones between 5550 and 5850 m asl, together with their degree of cell wall lignification, ages and sizes (min = minimum, max = maximum, stdev = standard deviation).

		Plant size (cm)				Plant age (years)				Degree of cell wall lignification (DCWL)			
Elevation (m asl)	No plants	min	mean	max	stdev	min	mean	max	stdev	min	mean	max	stdev
5850	52	0.2	3.1	11.2	2.9	11.0	29.8	63.0	13.9	8.7	34.3	55.4	13.0
5800	52	0.1	3.7	19.3	4.4	11.0	34.8	73.0	15.3	3.9	41.1	64.0	13.3
5700	43	0.0	7.0	33.1	8.8	11.0	29.3	64.0	14.8	34.1	59.7	88.3	14.4
5630	21	0.3	4.0	18.7	4.4	11.0	28.9	57.0	11.9	40.4	69.4	94.5	13.6
5550	39	0.1	0.9	3.7	0.8	11.0	23.8	40.0	7.3	43.2	68.6	98.3	12.7

Possible relationships between the size, age and DCWL of all 207 plants and elevation, which is a direct proxy for temperature, were tested by fitting a piecewise structural equation model (SEM) to the various input variables (Lefcheck 2016). We formulated a hypothetical causal model and fitted SEMs containing linear models for DCWL, plant size, plant age, and elevation. We expected a negative effect of elevation/temperature on lignification. However, as lignification may increase with age and overall plant size, and bigger and older plants could be more lignified and overrepresented at lower elevations, we tested the effect of elevation/temperature directly on DCWL and indirectly via plant size and age. The final SEM was obtained by iterative including/removing explanatory variables, which were centred and standardised before the analysis. We further calculated a variance inflation factor (VIF) to assess whether multi‐collinearity affected parameter estimates, and only variables with VIF < 5 were included in the final SEM. Fisher's C evaluated the goodness‐of‐fit for the whole model, and Nagelkerke pseudo‐*R* ^2^ values were reported to show the explained variance (Lefcheck 2016). SEMs were fitted by the ‘piecewiseSEM’ package version 2.3 (Lefcheck 2016), and VIFs were computed by the ‘car’ package version 3.0‐12.

## 3 Results and Discussion

The upper distribution limit of mature and healthy *P. pamirica* herbs was found at 5850 m asl (Figure 1B). Being home to one of the world's highest‐occurring vascular plants, the north‐western spur of the Himalayas provides ideal conditions to assess the functioning and productivity of alpine herbs under extreme cold temperatures. Mean annual precipitation totals are estimated to vary between 150 and 250 mm, and the mean soil moisture content during the growing season ranges between 15% and 25%, without any substantial changes along the elevational gradient. Although all cross‐sections represent mature and active stem parts at the root collar, our double‐stained thin sections reveal different tissue forms and cell functional types (Figure 1B and Supporting Information: Figures S2 and S3). Since dead and developing cells, disturb fractions and the cambium were excluded from the image analysis, we consider our DCWL estimates along the elevational gradient to be unbiased and comparable. Starch‐storing parenchyma cell walls stain blue, while DCWL may vary considerably between fibre cells and conducting vessels that provide structural and hydraulic functions, respectively. Despite its herbaceous nature, *P. pamirica* has a secondary cambium that produces xylem cells and should therefore be classified as a ‘woody’ plant. Our samples usually exhibit a thick bark, large cortex and xylem with much living parenchyma cells that contain a high soluble carbohydrate concentration as frost protection (Dolezal et al. 2016; Chlumská et al. 2022).

**Figure 2:**
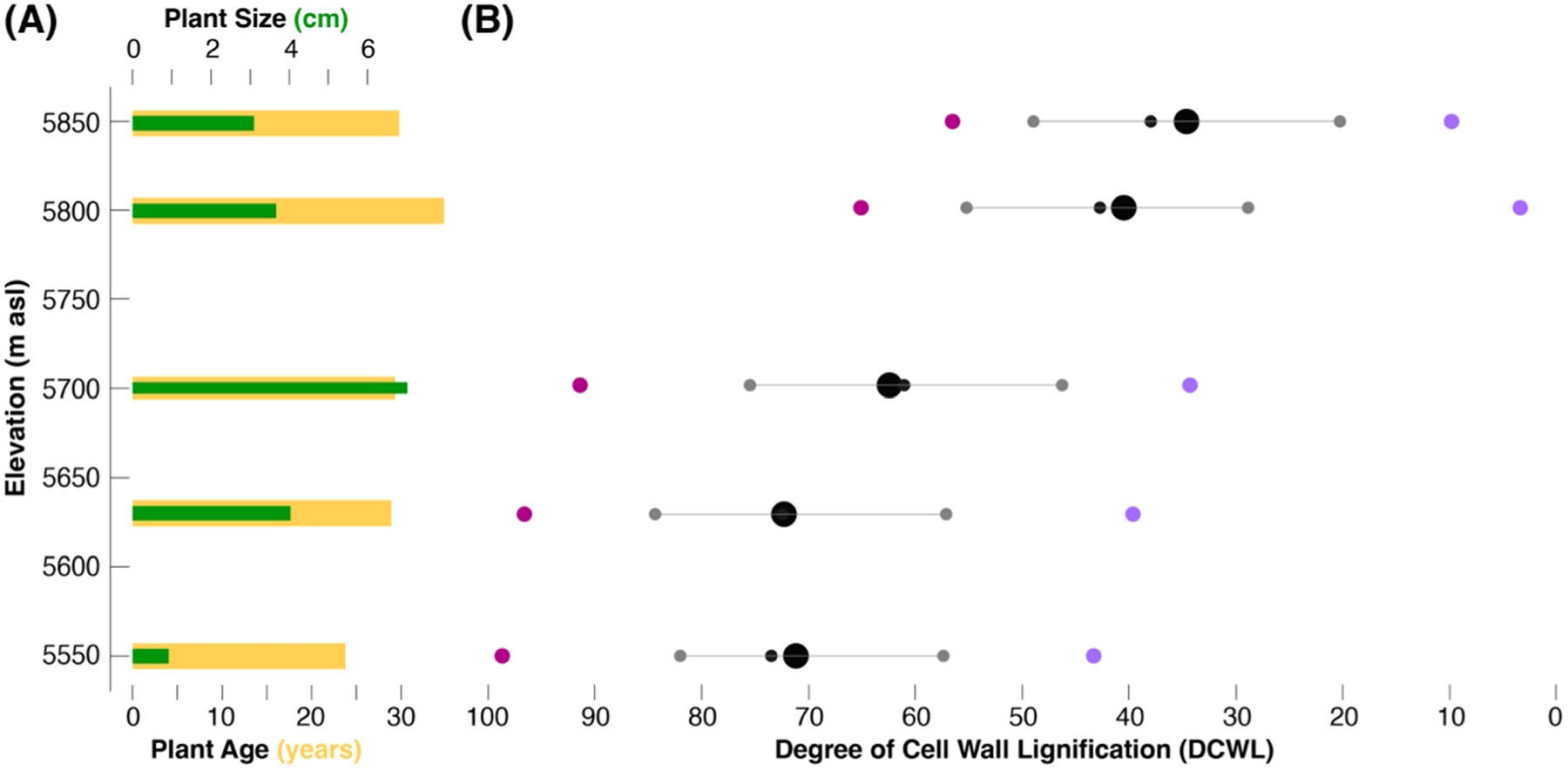
Changes in age, size and lignification of *Potentilla pamirica* with elevation. (A) Average age and size (in years and centimetres) of *Potentilla pamirica* at five elevational zones between 5550 and 5850 m asl (see Supporting Information: Figure S4 for the age and size of all individual 207 plants relative to elevation). (B) Mean and median (large and small black dots) of the degree of cell wall lignification (DCWL) of *Potentilla pamirica* at five elevational zones between 5550 and 5850 m asl (see Supporting Information: Figure S5 for the DCWL of individual plants), together with the minimum and maximum values (purple and pink), and 1 standard deviation (grey). [Color figure can be viewed at wileyonlinelibrary.com]

Intriguingly, both the size and age of *P. pamirica* are not decreasing with increasing elevation (Figure 2A and Supporting Information: Figure S4). The smallest and youngest herbs were collected at the lowest sampling zone around 5550 m asl and the tallest and oldest specimens were found in the middle or upper part of the elevational gradient (Table 1). Despite unprecedented recent summer warming across the Northern Hemisphere extra‐tropics (Esper, Torbenson, and Büntgen 2024), the lack of recruitment at the highest sampling sites could result from range‐pinning in the presence of strong Allee effects (Allee 1931), that is, species are not expanding at the edge of suitable habitats (Courchamp, Berec, and Gascoigne 2008). Commonly accepted in population/behaviour biology and ecology, the concept of positive density dependence has rarely been applied in treeline research (Büntgen et al. 2024), where empirical evidence should be combined with conceptual thinking.

**Figure 3:**
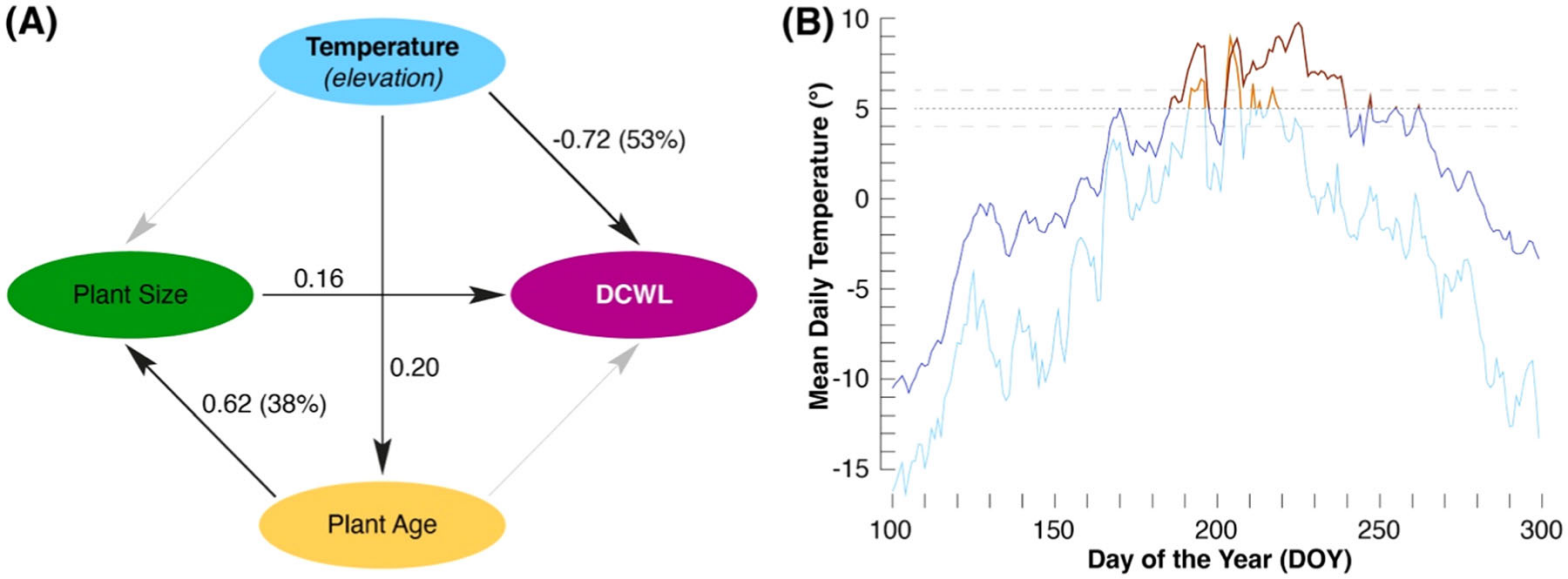
Temperature controls on lignin biosynthesis. (A) Structural equation model for temperature (elevation), plant age, plant size, and the degree of cell wall lignification (DCWL) in 207 *Potentilla pamirica* herbs that were collected between 5550 and 5850 m asl (see Table 1 for data characteristics). Black arrows describe significant correlations (*p* < 0.01), with percentages referring to explained variance in the model (Fisher's C = 0.108, *df* = 2, *p* = 0.947). (B) Mean daily soil (i.e., root zone) temperatures measured at 5550 and 5850 m asl (dark and light blue), with dark and light red lines referring to daily means > 5°C (see Table 2 for daily extremes and Supporting Information: Figures S6–S8 for full annual cycles). [Color figure can be viewed at wileyonlinelibrary.com]

Contrary to the relatively stable distribution of plant sizes and ages, the DCWL significantly (*r *= −0.73; *p* < 0.01) decreased with increasing elevation (Figure 2B and Supporting Information: Figure S5). While the mean DCWL of 60 herbs from the two lowest elevational zones is 69% (Table 1), this value drops by more than half for the highest samples (34%). Since elevation is an inverse proxy for temperature, our study provides strong evidence for a thermal control on the lignin biosynthetic pathway in the secondary cell walls of *P. pamirica* under extreme cold temperatures. The DCWL is predominantly defined by temperature (*r* = −0.73), but plant size and age are not associated with DCWL (Figure 3A). However, plant size is significantly (*p* < 0.01) affected by plant age, and the root collars of larger herbs tend to be more lignified than those of smaller specimens (*r* = 0.14). The striking relationship between temperature and lignin appears particularly reasonable given the extreme cold climate conditions along the entire elevational gradient (Figure 3B and Supporting Information: Figures S6–S8). Only 52 and 41 days experienced mean root zone and surface air temperatures above 5°C at 5550 m asl (Table 2), and these numbers dropped to 15 and 25 at 5850 m asl, respectively. Mean annual root zone temperatures are 5.6°C colder at the highest compared to the lowest sampling zone (Supporting Information: Figure S8A), and this value decreases to 1.4°C for surface air temperatures (Supporting Information: Figure S8B). The mean surface air temperature during 25 continuous days above ‘Biological Zero’ (> 5°C) is 7.3°C at the lowest sampling site and drops to 5.6°C for 12 successive days at the highest site (Supporting Information: Figure S6). Mean root zone temperature during 37 continuous days above ‘Biological Zero’ is 7.7°C at the lowest sampling site and drops to 7.5°C for only five successive days at the highest site (Supporting Information: Figure S7). While the observed difference in surface air temperature between the lowest and highest sampling zones reflects a lapse rate of circa 0.5 K per 100 m elevation, the decline in root zone soil temperature along the sampling gradient is even larger.

**Table 2:** Daily temperature extremes. Number of days that minimum (min), mean and maximum (max) daily soil and air temperatures were above 0°, 3°, 5° and 7°C. Daily values are based on 15‐min measurements throughout the entire calendar year of 2014.

		> 0°C Min	> 0°C Mean	> 0°C Max	> 3°C Min	> 3°C Mean	> 3°C Max	> 5°C Min	> 5°C Mean	> 5°C Max	> 7°C Min	> 7°C Mean	> 7°C Max
**AIR**	**5850 m asl**	32	96	151	0	36	103	0	25	77	0	2	58
	**5550 m asl**	4	104	200	0	67	184	0	41	169	0	19	156
**SOIL**	**5850 m asl**	1	65	145	0	33	119	0	15	101	0	3	80
	**5550 m asl**	104	124	126	36	90	112	9	52	101	0	30	75

Although small alpine plants can benefit from microclimatic effects (Körner, Fajardo, and Hiltbrunner 2023a; Körner 2012), these advantages also cease with elevation, and it is simply too cold for any type of vascular plant to establish and survive above the upper end of our sampling gradient. Moreover, we argue that the observed lack of lignin is not because small herbs do not need more strength to grow for up to 30 years, but because too cold temperatures reduce their lignin biosynthesis pathway. This chemical rather than physiological theory would apply to taller trees first, as such lifeforms are earlier exposed to colder air temperatures and therefore benefit less from daytime surface warming. The xylem of larger shrubs and trees is, however, always lignified because tall upright growing plants would not exist otherwise. Although certain shrub and herb species can establish and survive above the treeline, the upper and poleward distribution limits of smaller lifeforms also follow isotherms (Crivellaro et al. 2022; Büntgen et al. 2023). While we see evidence for a thermal threshold under which the lignin deposition stops, there is no indication for nonstructural carbohydrates to limit plant metabolism under extreme cold conditions.

## 4 Conclusions and Outlook

We performed a systematic analysis of the age, size and degree of stem cell wall lignification of 207 alpine herbs (*P. pamirica*), which were collected between 5550 and 5580 m asl on the most north‐western extent of the Himalaya mountain range in Ladakh, India. Our combined dendrochronological and wood anatomical approach revealed a significant negative effect of elevation on lignification (*r* = −0.73), whereby lignification and elevation must be understood as direct proxies for plant strength and ambient temperature, respectively. While the age and size of herbs are significantly correlated (*r* = 0.63), both factors are independent of elevation (i.e., temperature) and also had no significant impact on the DCWL.

Our findings from one of the world's highest‐occurring vascular plants add another line of evidence for a thermal control on lignin biosynthesis. This study also emphasises the possible role biochemical and biomechanical factors may play in defining the global treeline position. To provide further insights into possible drivers of the cold range limit of woody plant growth, we suggest exposing *Arabidopsis thaliana* (L.) Heynh and other model plants, including flax, to ephemeral cold spells. Further to the expansion of biogeographic studies to different species, lifeforms and life stages (Palosse et al. 2024), empirical evidence should be supplemented with conceptual thinking to improve eco‐physiological and global vegetation models. Finally, we argue that it is misleading to investigate trees below the upper treeline to understand why they do not establish and survive under colder conditions at higher elevations.

## Conflicts Of Interest

The authors declare no conflicts of interest.

## Supporting information

Supporting information.

## Data Availability

All data and codes used in this study are provided in the supplementary material.
