# Supplementary material for: Highest Occurring Vascular Plants from Ladakh Provide Wood Anatomical Evidence for a Thermal Limitation of Cell Wall Lignification
Source: Plant Cell Environ. 2024 Oct 24;48(2):1445–51. doi: 10.1111/pce.15221 (PMC11695796; doi:10.1111/pce.15221)
Supplement: Supplementary file 1 — Supporting information. [file PCE-48-1445-s001.docx]

**upplementary material**

Figures S1–S8

Code S1

Data S1


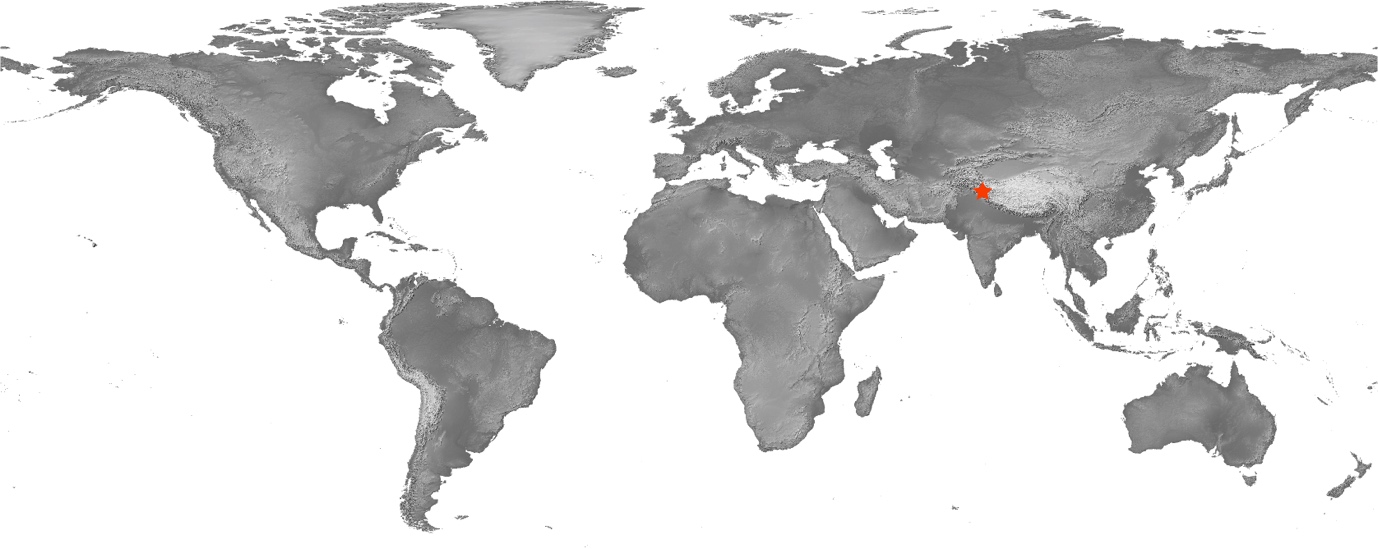


**Figure S1. Global context of our study site.** Red star refers to the location of our *Potentilla pamirica* sampling region near Chamser Kangri (6,622 m asl) in eastern Ladakh, north-western Himalaya (circa 32°59’ North and 78°29’ East).


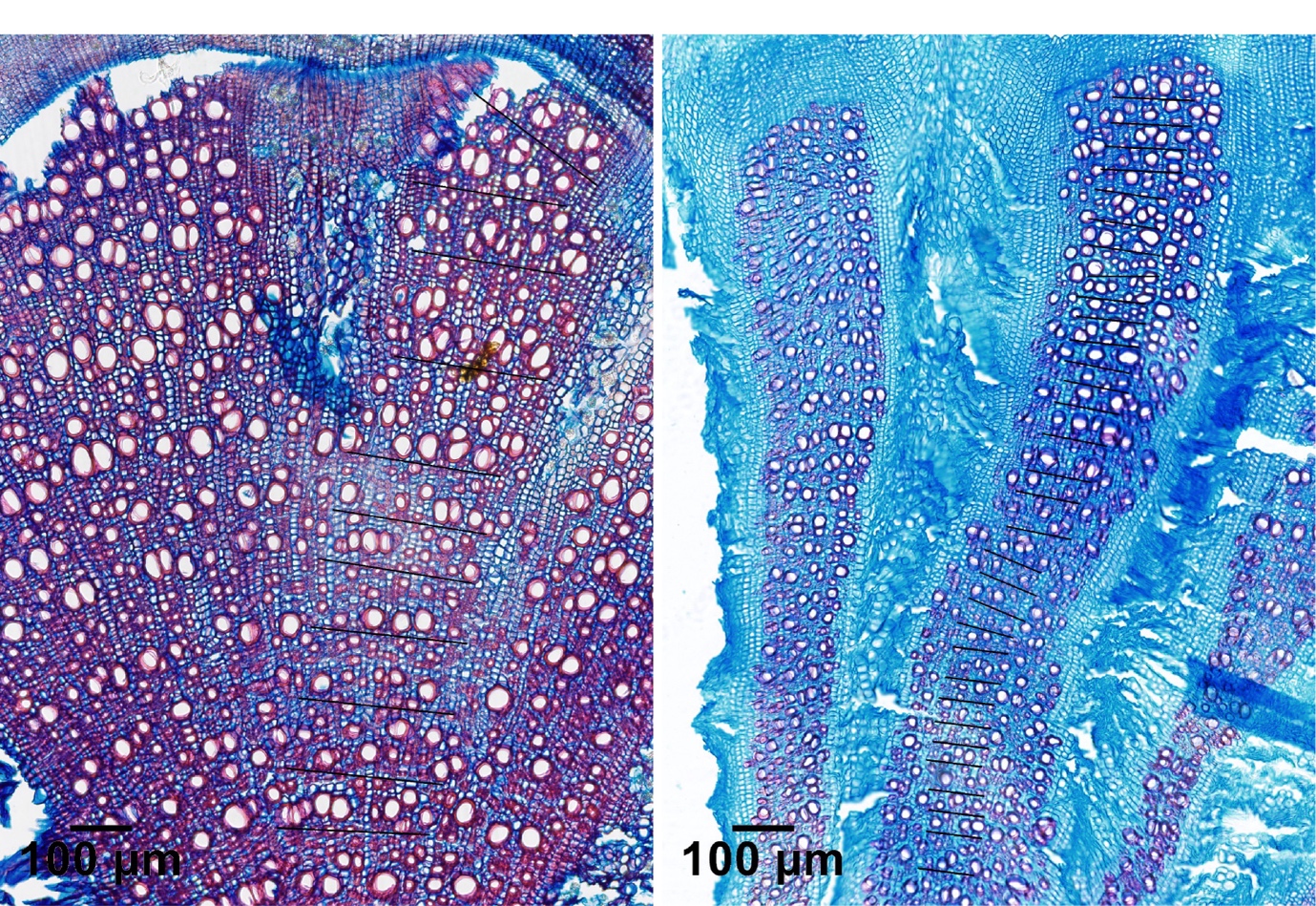


**Figure S2. Anatomical characteristics of *Potentilla pamirica* Wolf.** High-resolution microscopic images of two thin sections that were double-stained with Astra Blue and Safranin to reveal lignified (reddish) and less-lignified (blue) cell walls in the stems of *Potentilla pamirica* collected at 5,550 and 5,850 m asl in Ladakh (left and right images, respectively). The superimposed black lines refer to annual ring boundaries.


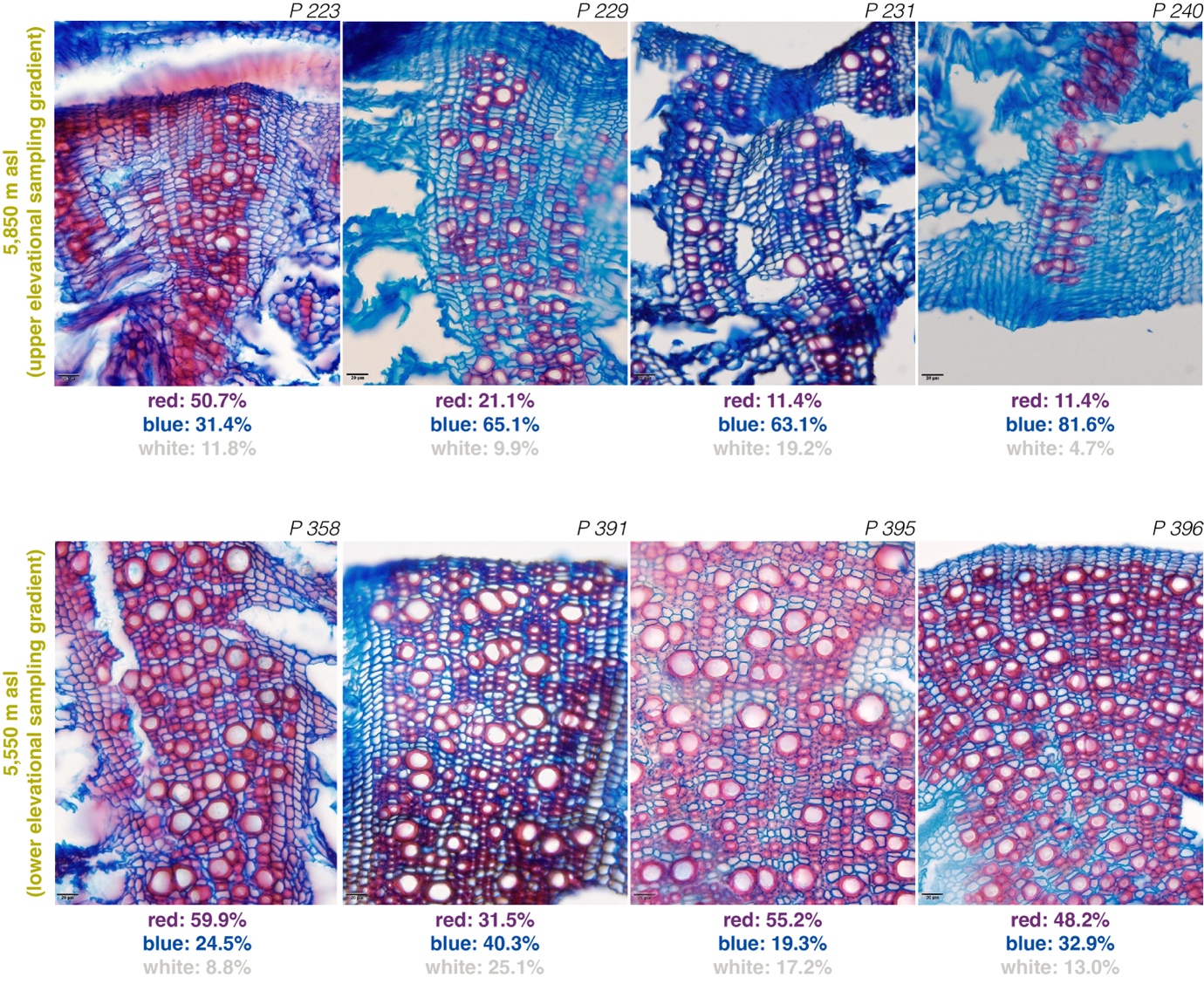


**Figure S3. Anatomical characteristics of *Potentilla pamirica* Wolf.** High-resolution microscopic images of a selection of thin sections that were double-stained with Astra Blue and Safranin to reveal lignified (reddish) and less-lignified (blue) cell walls in the stems of *Potentilla pamirica* collected at 5,550 and 5,850 m asl in Ladakh (lower and upper images, respectively). The percentage of red, blue and white area is indicated below the images.

**
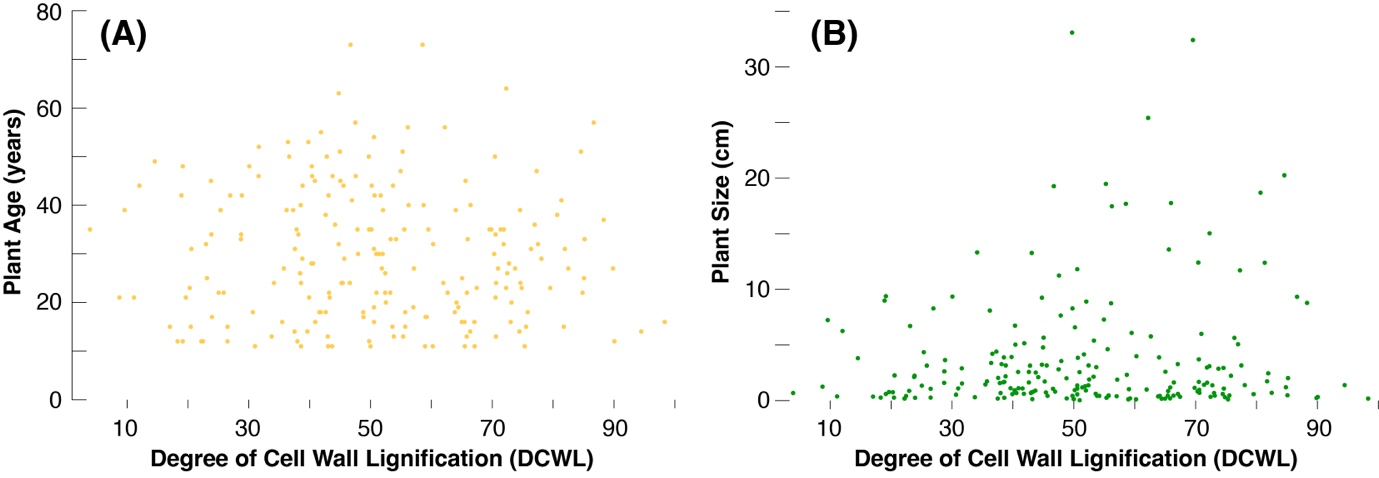
**

**Figure S4. Cell wall lignification and plant age/size.** (**A**) Relationship between the age (years) and degree of cell wall lignification (DCWL) of all 207 *Potentilla pamirica* herbs that were collected at five elevational zones between 5,550 and 5,850 m asl (see Table 1 for data characteristics). (**B**) Relationship between the size (cm) and degree of cell wall lignification (DCWL) of all 207 *Potentilla pamirica* samples.


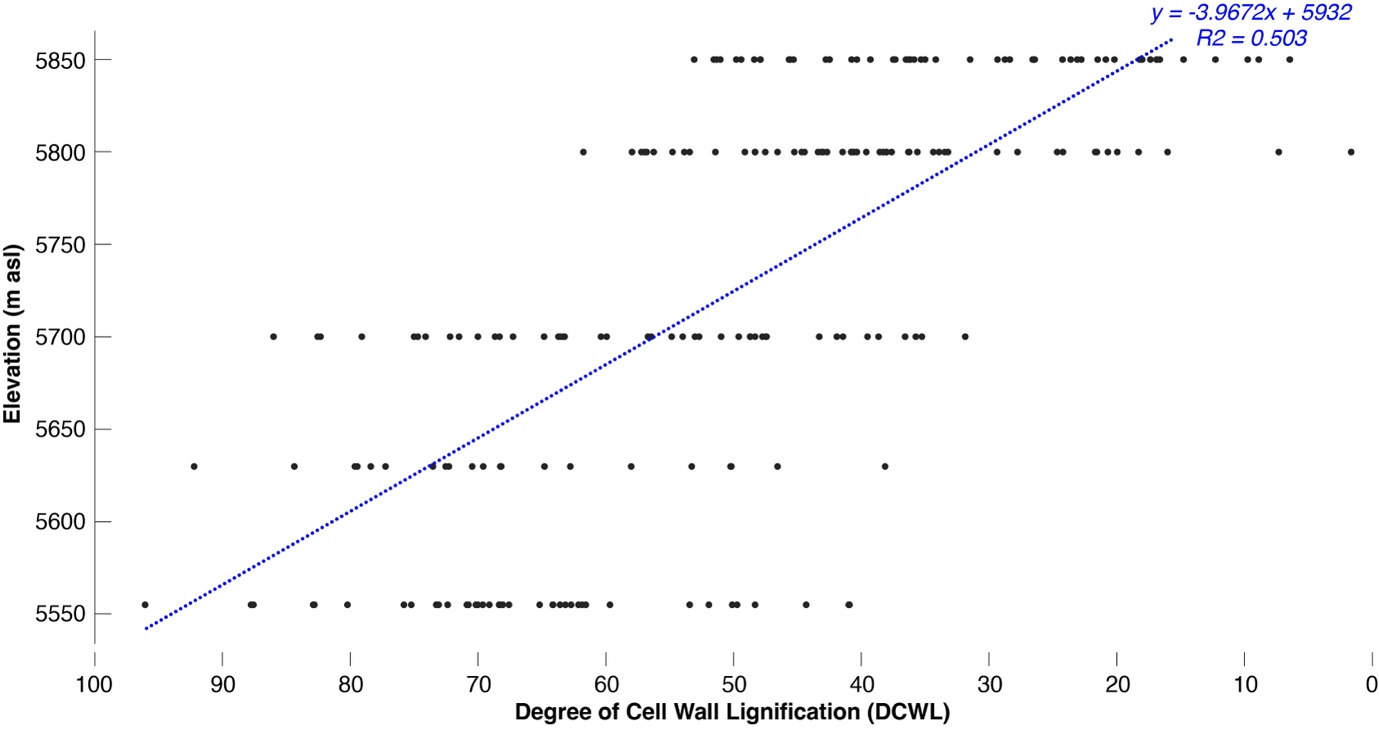


**Figure S5. Cell wall lignification and elevation.** The degree of cell wall lignification (DCWL) is shown for each of the 207 *Potentilla pamirica* herbs (black dots), which were collected at five elevational zones between 5,550 and 5,850 m asl (see Table 1 for data characteristics). Blue dashed line shows a significant linear trend across all 207 values (see formular at the upper right).


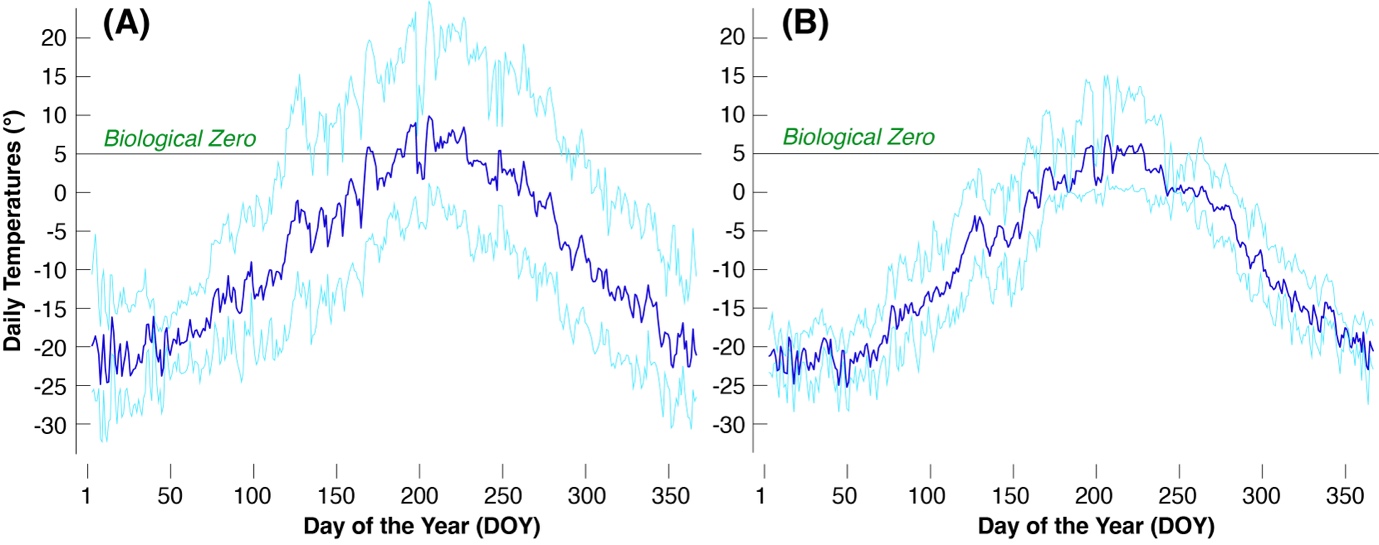


**Figure S6. Annual air temperature cycle.** Daily minimum, mean (dark blue), and maximum surface air temperatures measured at (**A**) the lowest and (**B**) the highest sampling zones at 5,550 and 5,850 m asl, respectively. The approximate thermal threshold of ‘Biological Zero’ is indicated at 5°C.


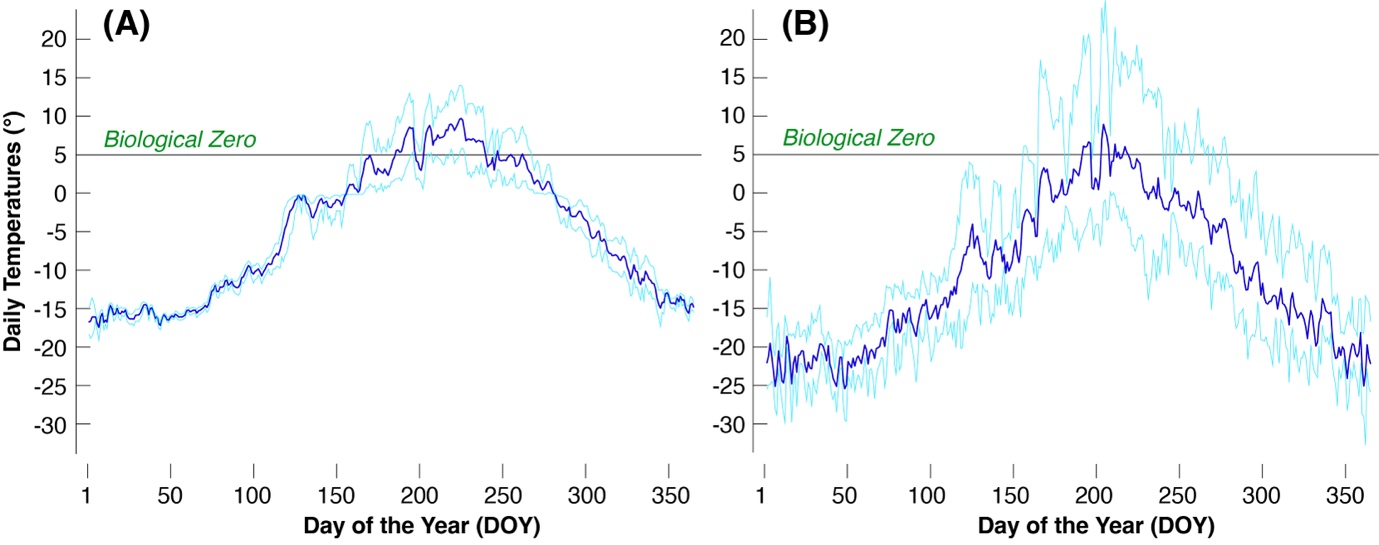


**Figure S7. Annual soil temperature cycle.** Daily minimum, mean (dark blue), and maximum root zone soil temperatures measured at (**A**) the lowest and (**B**) the highest sampling zones at 5,550 and 5,850 m asl, respectively. The approximate thermal threshold of ‘Biological Zero’ is indicated at 5°C.


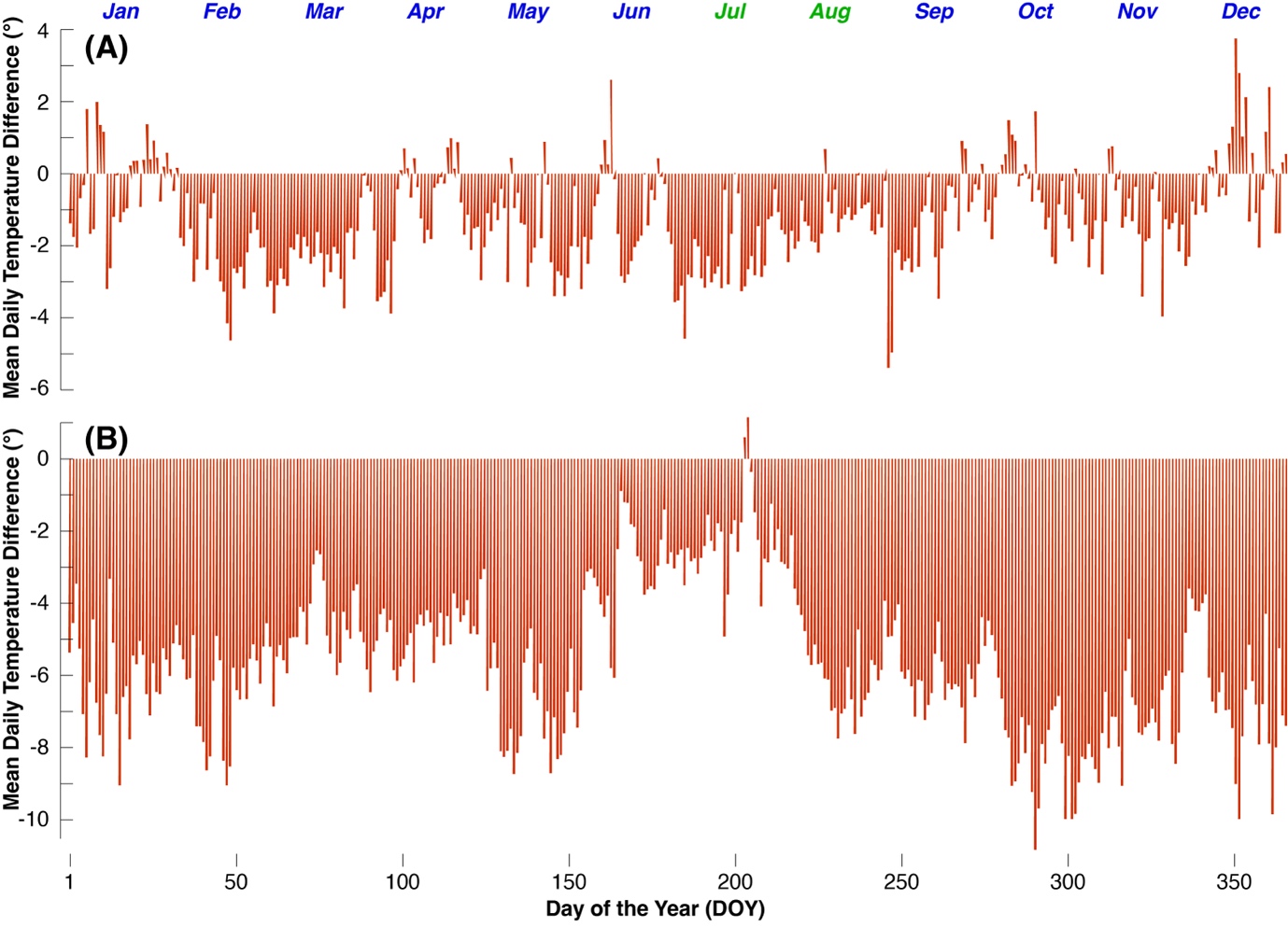


**Figure S8. Temperature differences with elevation.** (**A**) Differences in daily mean surface air temperature between the lowest and highest sampling zones at 5,550 and 5,850 m asl (high minus low), with an annual mean difference of -1.3°C (calculated over 365 days). (**B**) Differences in daily mean root zone soil temperature between the lowest and highest sampling zones at 5,550 and 5,850 m asl (high minus low), with an annual mean difference of -5.6°C (calculated over 365 days).
